# Evaluating the Early Impact of the COVID-19 Pandemic on Sports Surgery Fellowship Education

**DOI:** 10.7759/cureus.12943

**Published:** 2021-01-27

**Authors:** Peter R Swiatek, Joseph Weiner, Bejan A Alvandi, Daniel Johnson, Bennet Butler, Vehniah Tjong, Michael A Terry

**Affiliations:** 1 Department of Orthopaedic Surgery, Northwestern University Feinberg School of Medicine, Chicago, USA

**Keywords:** sports surgery, fellowship, education, covid-19, coronavirus, orthopaedic surgery

## Abstract

Purpose

The COVID-19 pandemic forced many hospitals to cancel elective surgeries to minimize the risk of viral transmission and ensure the availability of vital health resources. The unintended consequences of this action on the education and training of orthopaedic sports surgeons are unknown. The purpose of this study is to measure the impact of COVID-19 on orthopaedic sports surgery fellows, their education and training, and their readiness for practice.

Methods

A comprehensive survey was created and distributed to all U.S. fellows and fellowship directors registered with the American Orthopaedic Society for Sports Medicine. Responses were collected between April 22, 2020, and May 5, 2020.

Results

Fifty-one sports fellows and twenty-nine sports fellowship directors completed the survey. Over 80.4% of fellows reported a greater than 50% decrease in the case volume since the cessation of elective cases. Average hours worked per week decreased by 58.2% during the pandemic. Fellows reported completing an average of 324.6 ± 97.4 cases prior to the COVID-19 crisis and 86.0% expected to complete at least 11% to 25% fewer cases by graduation compared to previous fellows. 87.5% of fellows were not concerned about their ability to complete their fellowship training but more than one-third of fellows voiced concerns to their fellowship directors regarding their readiness for independent practice. Fellowship directors were generally not concerned that COVID-19 would prevent their fellows from completing the fellowship. At least 54.2% are somewhat concerned about the impact of COVID-19 on their future job opportunities.

Conclusions

The COVID-19 pandemic has universally affected work hours and case volume of sports fellows. Nevertheless, most sports fellows feel prepared to enter practice and are generally supported by the confidence of their fellowship directors. The results of this survey emphasize the importance of the fellowship year in sports training and highlight the future of online education and simulation as useful adjuncts.

## Introduction

The COVID-19 pandemic has emerged as a disastrous global health crisis [1,2]. Patients infected with COVID-19 placed an enormous strain on health care systems around the world [3]. Meeting these requirements in severely affected regions has proven demanding [4]. To address the transmission risk and help reallocate critical resources, many United States hospitals and surgery centers postponed elective surgeries in March 2020 [5].

Despite the interruption of elective surgery, orthopaedic departments across the United States have continued to provide urgent and emergent surgical services [6]. Although numerous editorials and society-level guidelines have been published to address the impact of COVID-19 on orthopaedic care [7-9], little is known about the effects of the pandemic on sports fellowship education.

Over the past several decades, fellowship training has become increasingly popular among orthopaedic surgeons [10]. The sports surgery fellowship is an intensive one-year of experiential learning during which trainees improve technical skills and gain valuable knowledge critical for diagnosing and managing sports-related conditions. Given the reported educational value of fellowship experience for sports surgery trainees [11,12] and the universal effect of COVID-19 on reducing elective orthopaedic surgery volume, we sought (1) to evaluate the impact of COVID-19 on sports fellow training and education, (2) to measure the effect of the pandemic on sports fellows’ readiness to enter practice, and (3) to discuss how COVID-19 has highlighted opportunities for improvement and innovation in sports fellow education.

## Materials and methods

Survey strategy and content

A team of board-certified orthopaedic sports surgeons and trainees designed the survey for current sports surgery fellows and fellowship directors during the 2019-2020 academic year. Questions were chosen for inclusion in the survey via the Delphi technique [13], in which attending-level surgeons reached consensus after several rounds of discussion. Two questionnaires were developed created using SurveyMonkey (San Mateo, California, USA) to target both sports surgery fellows and fellowship directors.

The survey targeted data such as geographic location, the timeline of elective surgery cancellation, impact on case-mix and volume, the effect on education, training, and job opportunities. Geographic location was broken down by region as defined by the United States Census Bureau [14]. The range of concern was measured using a Likert scale from one to five (1 = “non-concerned” to 5 = “extremely concerned”).

Survey circulation

The 36-question fellows’ questionnaire and 34-question fellowship directors’ questionnaires were circulated in English via email to American Orthopaedic Society for Sports Medicine (AOSSM) fellows (n=215) and fellowship directors (n=90). The survey remained open for two weeks (April 22, 2020 - May 5, 2020). Prior to the start of the survey, participants were informed that their contribution was entirely voluntary and that their results would be kept confidential and only analyzed and presented in aggregate in peer-reviewed journals, social media, or other web-based media outlets.

Statistical analysis

Data were collected from individual respondents, aggregated in Microsoft Excel (Redmond, Washington, USA), and analyzed. Means and percentages were calculated for rank-order and discrete data, respectively. Differences in continuous variables were assessed using student t-tests and differences in discrete variables were calculated using chi-square tests where applicable. Additionally, a univariate analysis was conducted to determine the impact of key independent variables, including fellowship regions/location, fellowship setting (i.e., urban, suburban, or rural), and the number of fellows in the program, on fellows’ overall concern regarding readiness to enter practice and their deployment to non-sports-related patient care roles. All analyses were executed using XLSTAT (Addinsoft, Paris, France). Differences were determined to be significant for p<0.05.

## Results

A total of 51 sports surgery fellows and 29 sports fellowship directors completed the survey, representing response rates of 23.7% and 32.2%, respectively. The majority of fellows (38/51, 74.5%) and half of the fellowship directors (15/29, 51.7%) reported training in urban locations. Most of the fellows represented programs with three or more fellows (28/51, 54.9%) whereas most of the fellowship directors represented programs with two or fewer fellows (19/29, 65.5%; Table 1).

**Table 1:** Survey respondent demographics

	Fellows		Fellowship directors	
	#	%	#	%
Fellowship region				
Northeast	16	31.4	6	20.7
Midwest	13	25.5	8	27.6
South	11	21.6	7	24.1
West	11	21.6	8	27.6
Fellowship setting				
Urban	38	74.5	15	51.7
Suburban	11	21.6	13	44.8
Rural	2	3.9	1	3.5
Number of fellows in the program				
1	7	13.7	9	31
2	16	31.4	10	34.5
3	7	13.7	4	13.8
4	5	9.8	3	10.3
5	8	15.7	3	10.3
6	3	5.9	0	0
7	1	2	0	0
8	2	3.9	0	0
9	2	3.9	0	0
Total respondents	51	100	29	100

Impact of COVID-19 and program response

All sports fellowship programs began enacting precautionary measures for COVID-19 prior to April 2020. The majority of fellows reported taking precautions between March 15 and 31, 2020 (37/51, 72.6%) compared to most directors, who reported taking precautions prior to March 15, 2020 (16/29, 55.2%). All respondents, with the exception of two sports fellows, reported cancellation of elective cases at the time of the survey. Most fellows (41/51, 80.4%) saw a decrease in the case volume by more than 50%. Prior to cessation of elective cases, sports fellows worked an average of 51.0 ± 11.3 hours per week. Afterwards the COVID-19 shutdown, mean hours worked per week dropped to 22.9 ± 15.9 hours, representing a decrease of 28.2 ± 15.9 mean hours per week (<0.0001). Eleven of 51 fellows (21.6%) reported being deployed to non-sports surgery roles, including four fellows who were assigned to manage COVID-19 units. In a univariate analysis, there was no difference in rates of re-deployment to non-sports surgery roles based upon fellowship region (p=0.57), fellowship setting (p=0.7), and the number of fellows in a program (n=0.55). Attending staff, trainees, or ancillary staff tested positive for COVID-19 at 48% (24/50) of fellows’ programs. Most (45/51, 90.0%) were satisfied with current COVID-19 precautions and discussed the impact of COVID-19 on training at least weekly (36/51, 75.0%; Table 2).

**Table 2:** COVID-19 response ^a^n=50 respondents; ^b^n=48 respondents; ^c^n=24 respondents; ^d^n=23 respondents.

Fellows			Fellowship directors		
	#/Mean	%/± SD		#/Mean	%/± SD
Start date of COVID-19 precautions			Start date of COVID-19 precautions		
Prior to March 1	0	0.0	Prior to March 1	1	3.5
March 1-14	14	27.5	March 1-14	15	51.7
March 15-31	37	72.6	March 15-31	13	44.8
April 1 to present	0	0.0	April 1 to present	0	0.0
No changes made	0	0.0	No changes made	0	0.0
Elective cases cancelled			Elective cases cancelled		
Yes	49	96.1	Yes	29	100.0
No	2	3.9	No	0	0.0
Decrease in overall institution case volume	51		Decrease in overall institution case volume		
0-25%	7	13.7	0-25%	2	6.9
26-50%	3	5.9	26-50%	2	6.9
51-75%	8	15.7	51-75%	3	10.3
>75%	33	64.7	>75%	22	75.9
Mean hours worked per week pre-COVID (n=51)	51.0	11.3	Mean hours fellows worked per week pre-COVID (n=29)	51.1	11.5
Mean hours worked per week post-COVID (n=50)	22.9	15.9	Mean hours fellows worked per week post-COVID (n=29)	21.3	12.9
Assisting with non-sports-related care			Fellows assisting with non-sports-related care		
Yes	11	21.6	Yes	7	24.1
No	40	78.4	No	22	75.8
Satisfied with current precautions^a^			Satisfied with current precautions^c^		
Yes	45	90.0	Yes	23	95.8
No	5	10.0	No	1	4.2
Frequency of COVID communication with fellowship director^b^			Frequency of COVID communication with fellow^d^		
Never	0	0.0	Never	0	0.0
Bi-weekly	12	25.0	Bi-weekly	6	26.1
Weekly	31	64.6	Weekly	7	30.4
Daily	4	8.3	Daily	10	43.5
Multiple times a day	1	2.1	Multiple times a day	0	2.1
Staff testing positive for COVID^a^			Staff positive for COVID^c^		
Attending staff	9	18.0	Attending staff	3	12.5
Fellow/resident	8	16.0	Fellow/resident	2	8.3
Ancillary staff	7	14.0	Ancillary staff	3	12.5
None	28	56.0	None	16	66.7
Prefer not to answer	9	18.0	Prefer not to answer	2	8.3
			Decision making for non-emergent surgeries^c^		
			Attending decision	7	29.2
			Service guidelines	5	20.8
			Hospital/OR guidelines	14	58.3
			State guidelines	8	33.3
			National guidelines	4	16.7
			Standardized scoring system to determine case priority^c^	9	37.5

The majority of fellowship directors (25/29, 86.2%) reported a decrease in the case volume by more than 50%. Their fellows worked an average of 51.0 ± 11.5 hours prior to the COVID-19 shutdown and subsequently worked 21.3 ± 12.9 hours during the height of the pandemic, which represented a 29.8 ± 13.1-hour decrease (p<0.0001). Seven directors reported that their fellows were re-deployed to non-sports surgery roles, including orthopaedic urgent care and trauma services. Attending staff, trainees, or ancillary staff tested positive at 33.3% of directors’ programs. Nearly, all directors were satisfied with current precautions taken at their institution (23/24, 95.8%). According to fellowship directors, the distinction between “emergent/urgent” cases versus “non-emergent” cases was primarily defined by hospital-specific guidelines (14/29, 58.3%) and state guidelines (8/24, 33.3%). Only nine fellowship directors used a standardized scoring system to determine case priority (9/24, 37.5%).

Case variety and volume

**Figure 1:**
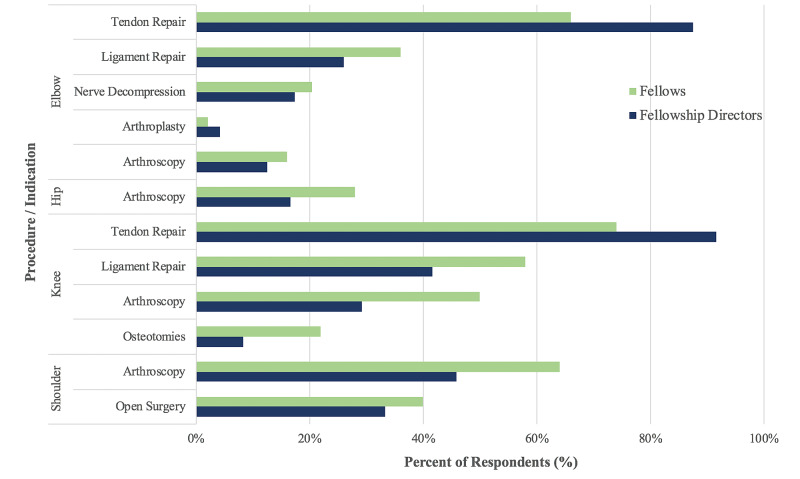
Case-mix of sports cases occurring during the COVID-19 pandemic. The most common elbow, hip, knee, and shoulder cases occurring during the cessation of elective cases. All numbers are self-reported by sports fellows and fellowship directors.

On average, fellows completed 324.6 ± 97.4 cases prior to the COVID-19 shutdown, and the majority (43/51, 86.0%) expected to complete at least 11% to 25% fewer cases by graduation compared to fellows from the previous years. Nearly, all fellowship directors reported similar expectations (23/24, 95.8%). Fellows reported distal biceps tendon repair (33/51, 66.0%), knee extensor tendon repair (37/51, 74.0%), and shoulder arthroscopy (32/51, 64.0%) as the most common sports cases still occurring at their institutions. Their responses mimic those of fellowship directors whose programs were still surgically treating patients with elbow tendon repairs (18/24, 75.0%), knee extensor tendon repairs (22/24, 91.7%), and shoulder arthroscopy (11/24, 45.8%; Figure 1). Fellows (29/51, 58.0%) and fellowship directors (10/24, 41.7%) also reported that ligamentous reconstructions after knee injuries were occasionally occurring at their institutions (Table 3).

**Table 3:** Case volume/mix

	Fellows (n=51)		Directors (n=24)	
	#/Mean	%/± SD	#/Mean	%/± SD
Cases personally completed prior to COVID-19 Shutdown	324.6	97.4	n/a	n/a
Estimated percent decrease in yearly case volume vs last year				
0-10%	7	14	1	4.2
11-25%	35	70	18	75
26-50%	7	14	5	20.8
>50%	1	2	0	0
Elbow cases still occurring				
Arthroscopy	8	16	3	12.5
Tendon repair	33	66	21	87.5
Ligament repair	18	36	6	26.1
Arthroplasty	1	2	1	4.2
Nerve decompression	10	20.4	4	17.4
Other				
Hip cases still occurring				
Arthroscopy	14	28	4	16.7
Other				
Knee cases still occurring				
Ligamentous reconstruction	29	58	10	41.7
Arthroscopy	25	50	7	29.2
Tendon repair	37	74	22	91.7
Osteotomies	11	22	2	8.3
Other				
Shoulder cases still occurring				
Arthroscopy	32	64	11	45.8
Open surgery	20	40	8	33.3
Arthroplasty	13	26	4	17.4
Other				

Readiness for practice

**Figure 2:**
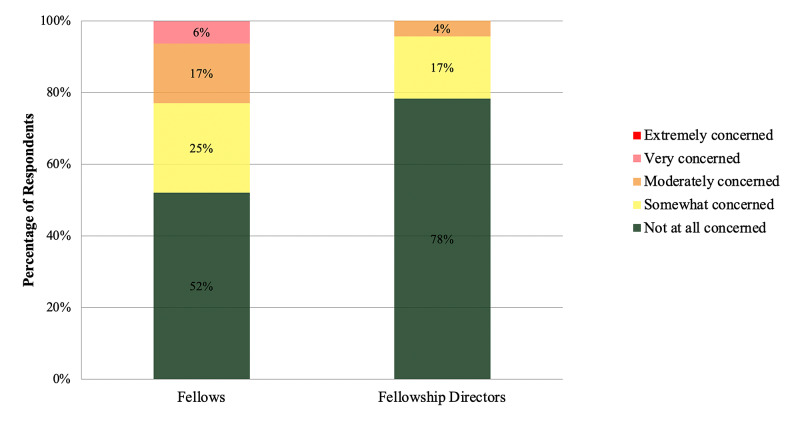
Self-reported concern regarding fellow readiness for practice. Fellow and fellowship director self-reported concern regarding fellow preparedness to begin practice after fellowship graduations.

Fellows were generally not concerned that COVID-19 would limit their ability to complete fellowship (42/48, 87.5%). Prior to the pandemic, fellows expressed very low concern regarding their ability to enter practice after fellowships (1.54 ± 0.8, 1=not concerned, 5=extremely concerned). There was a trend toward increased concern during the shutdown of elective cases, but the increase did not reach statistical significance (1.77 ± 1.0, D0.23 ± 0.4, p=0.204; Figure 2). Fellows believed that they would be ready to begin practice if elective case began in May (48/48, 100%), June (46/48, 95.8%), or July (36/48, 75.0%), and more than one-third of fellows voiced concerns about their fellowship directors regarding their preparedness to start practice (Table 4). In a univariate analysis, levels of self-reported concern and incidence of reporting concern to the fellowship director were not associated with fellowship region (p=0.18 and p=0.06), fellowship setting (p=0.3 and p=0.06), or the number of fellows in a program (0.34 and 0.55).

**Table 4:** Fellow preparedness vs fellowship director assessment of preparedness

Fellow (n=48)			Fellowship director (n=23)		
	#/Mean	%/± SD		#/Mean	%/± SD
Will COVID limit fellowship completion?			Will COVID prevent your fellow from graduating?		
Yes	6	12.5	Yes	4	17.4
No	42	87.5	No	19	82.6
Concern regarding the ability to enter practice pre-COVID (1-not concerned, 5-extremely concerned)	1.54	0.8	Concern regarding the ability to enter practice pre-COVID (1-not concerned, 5-extremely concerned)	1.08	0.3
Concern regarding the ability to enter practice post-COVID (1-not concerned, 5-extremely concerned)	1.77	1	Concern regarding the ability to enter practice post-COVID (1-not concerned, 5-extremely concerned)	1.24	0.5
Prepared to start practice if elective cases resume in:			Fellow prepared to start practice if elective cases resume in:		
May	48	100	May	21	91.3
June	46	95.8	June	22	95.6
July	36	75	July	18	78.3
Voiced concern to fellowship director about preparedness	12	37.5	Fellow voiced concern about preparedness	8	35

Similar to fellows, fellowship directors were generally confident that COVID-19 would not prevent their fellows from graduating (19/23, 82.6%). Prior to the COVID-19 shutdown, fellowship directors were not concerned regarding their fellows’ ability to start practice after graduation (1.08 ± 0.3, 1=not concerned, 5=extremely concerned). There was a trend toward increased concern during the COVID-19 shutdown, but this did not reach statistical significance (1.24 ± 0.5, D0.16 ± 0.4, p=0.183). Fellowship directors believed that their fellows would be ready to begin practice if elective cases began in May (21/24, 91.3%), June (22/24, 95.6%), or July (18/24, 78.3%). Despite their relatively low concern, 35% of fellowship directors (8/23) reported that their fellows voiced concerns to them regarding readiness to start practice after fellowship.

Education and job opportunities

Fellows and fellowship directors reported changes to their sports curriculum, with the transition to a digital or virtual curriculum for didactics being most commonly reported by fellows (42/48, 87.5%) and directors (20/23, 87.0%). Most sports fellows were in contact with other sports fellows during the pandemic (34/48, 70.8%), but many did not feel connected to other fellows (20/48, 41.7%). Similarly, fellowship directors were in contact with other directors regarding fellow education (12/23, 52.2.%); however, 30.4% of directors felt disconnected from other directors (7/23). The majority of fellows (41/48, 85.4%) reported having jobs arranged prior to the start of the COVID-19 pandemic and most fellows (26/48, 54.2%) are at least somewhat concerned that their future job may be affected by the COVID-19 (Table 5).

**Table 5:** Fellow education and employment opportunities

Fellow (n=48)			Fellowship director (n=23)		
	#	%		#	%
Changes to the education curriculum			Changes to the fellow education curriculum		
Met less frequently (in-person or virtually)	10	20.8	Met less frequently (in-person or virtually)	3	13
Met more frequently (in-person or virtually)	20	41.7	Met more frequently (in-person or virtually)	6	26.1
Changed to a digital/virtual curriculum for didactic education	42	87.5	Changed to a digital/virtual curriculum for didactic education	20	87
Already was using a digital/virtual curriculum for didactic education	0	0	Already was using a digital/virtual curriculum for didactic education	1	4.4
Contact with other sports fellows during the pandemic			Contact with other fellowship directors about fellow education		
Yes	34	70.8	Yes	12	52.2
No	14	29.2	No	11	47.8
Felt connected to other fellows during the pandemic			Felt connected to other fellowship directors during the pandemic		
Yes	28	58.3	Yes	16	69.6
No	20	41.7	No	7	30.4
Job arranged prior to COVID					
Verbal commitment	8	16.7			
Received offer letter but had not signed	7	14.6			
Signed contract	26	54.2			
No	7	14.6			
Cancelled job interviews	9	22.9			
Concern regarding job start date or rescinding of job offer					
Very concerned	2	4.2			
Somewhat concerned	24	50			
Not concerned	16	33.3			
I do not have a job lined up yet	6	12.5			

## Discussion

The COVID-19 pandemic is not the first global health crisis to challenge U.S. health care systems. During the 20th century, there were several pandemics: the H1N1 Spanish flu of 1918, the H2N2 Asian flu of 1957, and the H3N2 Hong Kong flu of 1968 [15]. However, the COVID-19 pandemic crisis is the first to impact the U.S. since the expansion of orthopaedic fellowship education. Given that 90% of orthopaedic surgery residents pursue a post-graduate fellowship to refine their clinical acumen and surgical skill set prior to entering practice, the impact of COVID-19 on training is undoubtedly anxiety-provoking for many, including sports surgery fellows. Several recent editorials have discussed the impact of COVID-19 on orthopaedic trainee education [9,16,17]. For example, a group of spine surgery fellows in New York City described their experience of being re-deployed to non-spine-related patient care services and the impact the cancellation of all elective cases had on their training and educational objectives [17]. No study to date, however, has measured the impact of COVID-19 on sports surgery fellow training and education. We analyzed input from the AOSSM academic community, specifically 51 sports surgery fellows and 29 sports fellowship directors, to better understand the state of sports surgery training during the COVID-19 pandemic, to assess the effect of the pandemic on fellows’ preparedness for practice, and to discuss potential opportunities to improve sports surgery fellow education.

Our survey revealed that nearly all sports surgery programs saw a complete cessation of elective cases and a more than 50% work hour reduction beginning in mid-March 2020. Most fellows and fellowship directors anticipated that each fellow would graduate with 11% to 25% fewer cases compared to previous years’ trainees. To continue sports education during the COVID-19 shutdown, many programs transitioned their curriculum to virtual or web-based platforms similar to certain spine fellowship [17] and orthopaedic residency [9] programs. Authors from one orthopaedic residency program in New York City describe how their program was able to continue their standard conference schedule virtually while expanding educational opportunities to include presentations from other local programs and industry, lectures from American Hip and Knee Society FOCAL curriculum, and virtual reality (VR) experiences via headsets for residents at home [18]. Needless to say, COVID-19 has ignited the topic of online education for orthopaedic residents and fellows. Several authors even predict that online education may become a part of the “new normal” for orthopaedic resident and fellow education [16-18]. At this stage, however, the rapid rise in online education offerings and resources seems to be fragmented and somewhat unstructured. Moreover, hands-on experience is still widely considered the gold standard in surgical skills training and adjunctive learning modalities, such as sawbones and simulators, are used primarily to augment invaluable operating room experiences [19,20]. Nevertheless, we anticipate opportunities in the near future to pressure-test, standardize, and package pieces of the orthopaedic and sports curricula to supplement and reinforce current sports trainee education.

Despite the reduction in work hours and case volume, fellows and fellowship directors were only slightly concerned that COVID-19 would impact fellow readiness to start practice after graduation. Nearly, all fellowship directors expected their fellows to be prepared to graduate if elective cases resumed by June. Notably, however, 37.5% of fellow voiced concerns regarding their preparedness to their fellowship directors and 35% of fellowship directors reported hearing similar concerns from their fellows. The disconnect in concern voiced by the fellows and the assurance offered by the fellowship directors is likely due to the fact that many residents and fellows seek to gain their surgical self-confidence through experience and case volume [21]. Knowing that much of the sports literature promotes an association between surgeon volume and patient outcomes [22,23], many fellows may feel the need to prove their surgical skills to themselves prior to entering independent practice. Fellowship directors, on the other hand, maybe more focused on the demonstration of core competencies as criteria for graduating their fellows. Regardless, the consequence of COVID-19 on decreased case volume raises the question of whether a competency-based education model may be more effective than the current time-based training model for orthopaedic residents and sports fellows.

The current model for surgical training was established in 1889 by Dr. William Halstead and has remained relatively unchanged [24]. In the past decade, the academic orthopaedic community slowly began to introduce the concept of competency-based education [25]. The American Board of Orthopaedic Surgery, in cooperation with the Accreditation Council for Graduate Medical Education (ACGME), launched an initiative aimed at defining and assessing the essential clinical, surgical, and professional skills necessary for orthopaedic surgery residents to function as independent orthopaedic surgeons [26]. Despite the well-intentioned proposal, adoption of a competency-based orthopaedic education has been slow and met with resistance, owing primarily to the difficulty of standardizing assessments and the need for “buy-in” from all academic faculty [27]. We propose the impact of COVID-19 on sports surgery training highlights the importance of transitioning orthopaedic and sports fellow training to a robust competency-based model that ensures each fellow graduates with the clinical acumen and surgical skill set necessary to confidently pursue their professional orthopaedic practice.

Lastly, we identified that current sports surgery fellows are anxious about the current job market and future employment opportunities. At the time of this survey, 85.4% of sports fellows had at least received an offer letter or verbally committed to a job. However, widespread financial uncertainty across health care systems has led to furloughs [28] and hiring-freezes [29], leading more than half of sports fellows to be at least somewhat concerned about their future employment status. Although the job market of orthopaedic surgeons has historically been strong [30], only time will tell how this unprecedented crisis will affect employment success.

This survey-based study was not without limitations. First, although on par with expectations for an external survey, response rates were relatively low, representing 23.7% and 32.3% of all surveyed AOSSM fellows and fellowship directors, respectively. Low survey response rates may introduce biases that influence our ability to draw generalizable conclusions for all sports fellowship programs. Second, this survey was focused primarily on fellows’ preparedness to perform surgically in the operating room. The effect of COVID-19 on the outpatient clinical experience was excluded from this study. Next, we assessed the impact of COVID-19 on fellow work hours; however, the survey did not delineate exactly how fellows were spending their time (i.e., non-sports surgery patient care, research, other orthopaedic services, etc.). Lastly, the survey reports subjective data regarding clinical and surgical aptitude and did not directly measure the effects of the COVID-19 pandemic on clinical or surgical proficiency. Nevertheless, we believe the results of this survey provide meaningful insight into the impact of COVID-19 on sports fellowship training, the self-perceived readiness of current sports fellows to enter practice after graduation, and the opportunity to improve sports surgery education and training in the post-pandemic era.

## Conclusions

The impact of COVID-19 on health care systems across the world has been unprecedented. For sports surgery fellows, COVID-19 has directly affected work hours, case volume, case mix, and in-person education opportunities. Despite these specific challenges, sports fellows generally expect to be ready to enter practice after graduation, and their expectation is supported by the assurance of their fellowship directors. This survey also serves to highlight potential opportunities for innovation in online education and improvement in the sports fellowship education model.
